# Asymmetry-induced resistive switching in Ag-Ag_2_S-Ag memristors enabling a simplified atomic-scale memory design

**DOI:** 10.1038/srep30775

**Published:** 2016-08-04

**Authors:** Agnes Gubicza, Dávid Zs. Manrique, László Pósa, Colin J. Lambert, György Mihály, Miklós Csontos, András Halbritter

**Affiliations:** 1 Department of Physics, Budapest University of Technology and Economics, Budafoki ut 8, 1111 Budapest, Hungary; 2 MTA-BME Condensed Matter Research Group, Budafoki ut 8, 1111 Budapest, Hungary; 3 Physics Department, Lancaster University, Lancaster, UK

## Abstract

Prevailing models of resistive switching arising from electrochemical formation of conducting filaments across solid state ionic conductors commonly attribute the observed polarity of the voltage-biased switching to the sequence of the active and inert electrodes confining the resistive switching memory cell. Here we demonstrate stable switching behaviour in metallic Ag-Ag_2_S-Ag nanojunctions at room temperature exhibiting similar characteristics. Our experimental results and numerical simulations reveal that the polarity of the switchings is solely determined by the geometrical asymmetry of the electrode surfaces. By the lithographical design of a proof of principle device we demonstrate the merits of simplified fabrication of atomic-scale, robust planar Ag_2_S memory cells.

As ongoing miniaturization reaches the fundamental limitations of silicon-based complementary metal-oxide-semiconductor (CMOS) technology, the demand for alternative material platforms delivering faster, smaller, yet highly integrable logical and memory units is increasing. Self-assembled nanostructures exhibiting tunable electrical properties are primary candidates. Conducting nanofilaments formed or destroyed by reversible solid state electrochemical reactions in ionic conducting media situated between metallic electrodes have demonstrated reproducible logical and non-volatile resistance switching random access memory (ReRAM) operations^1^,^2^,^3^,^4^,^5^,^6^,^7^,^8^,^9^,^10^,^11^,^12^,^13^,^14^,^15^,^16^. The resistance of such a two-terminal memristor^17^, is altered above a threshold bias (*V*_th_) of a few hundred mV. Nonvolatile readout is performed at *V* ≪ *V*_th_^18^.

Nanofilament formation in solid state electrolytes^4^,^7^,^16^,^19^,^20^,^21^,^22^,^23^,^24^,^25^,^26^,^27^,^28^,^29^,^30^,^31^,^32^,^33^,^34^,^35^ are commonly attributed to oxidation, electric-field-driven ionic migration and reduction, involving a positively charged active electrode supplying the mobile ions and a negatively charged inert electrode, where reduction can take place initializing the filament growth. At opposite polarity, the filament is dissolved. While offering extremely large *R*_OFF_/*R*_ON_ switching ratios, devices operated in this regime can only perform at reduced switching speeds due to their fundamental RC limitations. Once such a metallic nanofilament bridging the two electrodes is fully developed, smaller but orders of magnitude faster resistance changes can be observed as the filament diameter is modulated^15^,^18^,^36^,^37^,^38^,^39^,^40^. Our present study focuses on the latter regime.

The central question of our Letter concerns the role of the inert electrode and consequently, the polarity of the set/reset transitions. Depending on the ionic mobility and redox rates, nucleation and subsequent filament formation have been observed either at the inert or at the active electrode surfaces by *in-situ* methods^23^,^31^,^32^,^41^. After an initial nucleation phase further reduction takes place directly along the growing filament consisting of the elemental metal of the active electrode. Thus, we anticipate that resistive switching must also occur when both electrodes are fabricated from the active material. We propose that the polarity of the resistive switching is determined by the local inhomogeneity of the electric field, the latter reflecting the geometrical asymmetry of the electrode surfaces with particular emphasis on the narrowest region of the filament.

Single-component metallic junctions utilizing silver^42^ and aluminium^43^ break junctions exhibit reproducible resistive switching at cryogenic temperatures, both relying on atomic rearrangements due to electromigration. Similar experiments were also carried out using nanofabricated gold nanowires^44^ at room temperature. While the operation speed of these devices were (instrumentally) limited to 100 kHz, Ag-Ag_2_S-PtIr memristive nanojunctions were successfully demonstrated to exhibit nanosecond switching times^39^. However, the fabrication of such ultrafast, Ag_2_S based memory cells would be considerably simplified if a single metallic component could be utilized for both terminals of the devices. Here we demonstrate stable resistive switchings in metallic Ag-Ag_2_S-Ag nanojunctions created by an STM and alternatively, in a mechanically controllable break junction (MCBJ)^45^ setup. The observed set/reset transitions exhibit a uniform polarity with respect to the initial asymmetry of the junction geometry in agreement with our molecular dynamical simulations. The latter reveal the kinetics of filament growth and shrinkage under various boundary conditions by taking into account electric-field-driven activated ionic migration in the Ag_2_S matrix. Based on these findings a proof of concept all-Ag on-chip device fabricated by standard electron beam lithography is testified to reliable memory operations.

## Results and Discussion

**Figure 1:**
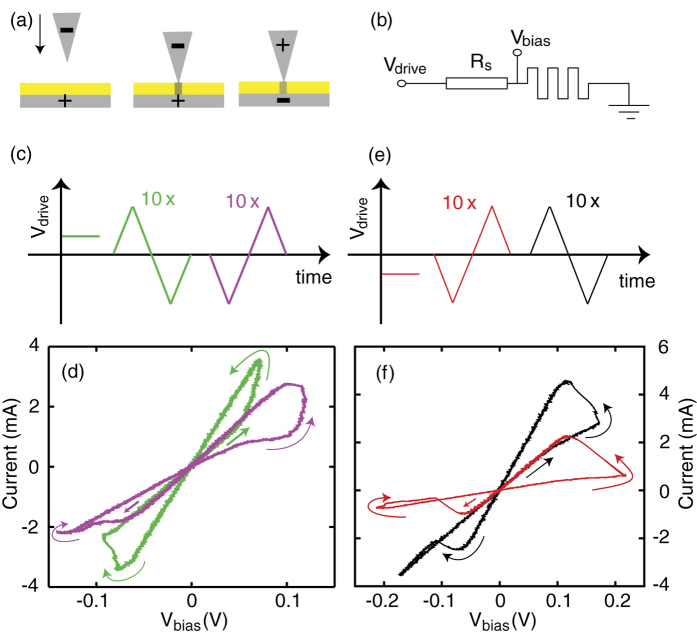
(**a**) The scheme of the I-V measurements performed in the STM setup. A constant bias approach of the tip is followed by voltage sweeps of alternating sign. By convention, a positive bias corresponds to a positive voltage applied to the Ag layer with respect to the STM tip. (**b**) The electrical circuit diagram of the biasing setup. (**c**) A typical sequence of the triangular *V*_drive_ signals of 2.5 Hz. The junction is established in the presence of a constant positive voltage of *V*_drive_ = 100 mV (green). Resistive switching behavior is investigated by a triangular *V*_drive_ starting with a positive polarity (green) which is reversed after 10 periods (magenta). (**d**) The corresponding hysteretic I-V traces exhibiting a uniform switching direction as indicated by the curved arrows. The straight arrows denote the initial configurations. (**e**) Approaching at a constant negative voltage of *V*_drive_ = −100 mV (red) followed by a reversed sequence of *V*_drive_ with respect to (**c**) (red and black). (**f**) The corresponding I-V traces reveal identical directions of the hysteresis loops to those in (**d**). The displayed device resistances are *R*_OFF_ = 38 Ω, *R*_ON_ = 20 Ω (green), *R*_OFF_ = 63 Ω, *R*_ON_ = 34 Ω (magenta), *R*_OFF_ = 256 Ω, *R*_ON_ = 51 Ω (red) and *R*_OFF_ = 49 Ω, *R*_ON_ = 23 Ω (black). *R*_S_ = 50 Ω and 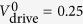 V.

Representative I-V traces obtained in the inherently asymmetric STM geometry are displayed in Fig. 1. In order to investigate the influence of the initial electroforming process on the direction of the observed resistive switching, measurements were performed on nanojunctions established with Ag tips, which were either negatively or positively charged during approaching the Ag_2_S thin films, as illustrated in Fig. 1(a). After forming a metallic contact, a triangular *V*_drive_ voltage signal of the same initial polarity was applied to record the I-V traces over 10 periods. This was followed by a reversed phase triangular *V*_drive_ of another 10 periods as indicated in Fig. 1(c,e). The corresponding I-V traces are exemplified in Fig. 1(d,f). It is to be emphasized that all the four hysteresis loops share the same direction of the resistive switchings, i.e., set (reset) transitions take place exclusively at positive (negative) biases on the Ag film, independently of the bias polarity on approaching as well as of the initial field direction during the voltage sweeps. This provides a strong experimental evidence that the polarity of the resistive transitions in the Ag_2_S layer is solely determined by the inhomogeneity of the local electric field in the vicinity of the conducting filament in accordance with the geometrical asymmetry of the surrounding Ag terminals. In order to gain a microscopic insight into the kinetics of field driven filament evolution upon such biasing cycles and to understand the observed, robustly uniform polarity of the resulting resistive switchings we performed atomic-scale numerical simulations taking all-Ag electrodes with various boundary conditions into account.

The simulations were carried out on a two-dimensional equilateral triangular lattice, where the lattice sites are either empty or occupied by a silver ion or atom. The time development is performed either by moving some of the silver ions or atoms to their neighboring empty site or by simulating a redox reaction, in which silver ions and atoms located at an electrode surface are exchanged. First the electrostatic potential is computed in each time step. This is followed by the calculation of a transition probability for each possible change. Finally the changes are executed with the calculated probabilities. The transition probabilities are computed as 
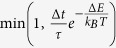
 where Δ*E* is the energy cost of the displacement or the redox reaction, 1/*τ* is the attempt rate and Δ*t* is the duration of the time step. The Δ*E* energy change depends on the participating atom’s or ion’s interaction with its neighbors. In case of silver ions it also depends on the electrostatic potential. The ion-ion and ion-atom interactions are parameterized close to room temperature in order to keep the silver ions sufficiently mobile. The atom-atom interaction is set to be strongly attractive enabling the growth of stable metallic branches which resist to thermal diffusion. Further technical details of the simulations are available in the Supplementary Information. We emphasize that the two-dimensional aspect of the above model along with the assumption of a triangular lattice and the phenomenological transition probabilities obviously cannot account for the rich variety of the microscopic details present in real Ag_2_S nanojunctions. Yet, the reduced computational requirements of such a simplified model allowed the analysis of several different parameter sets and boundary conditions and thus provided a deeper understanding on the actual tendencies of electric field driven filament evolution at asymmetric electrode configurations.

**Figure 2:**
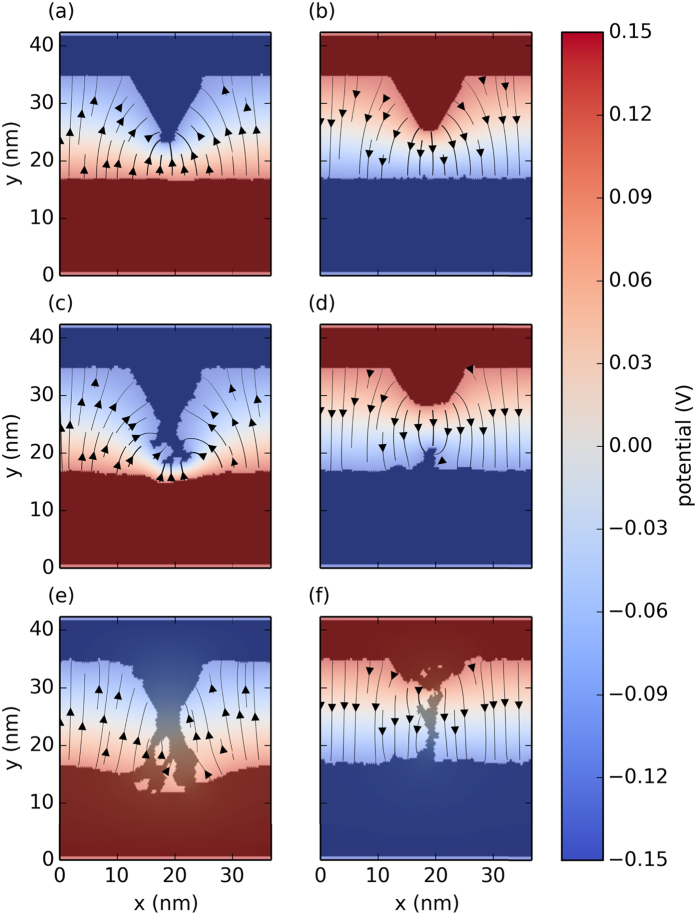
Snapshots of initial silver filament formation within the Ag_2_S layer at an asymmetric initial tip versus flat surface arrangement of the Ag electrodes. The semi-transparent color map indicates the electrostatic potential and the stream lines visualize the electric field direction and magnitude across the Ag_2_S layer. The left and right panels show the junction’s evolution for negative and positive tip potential *V*_bias_, respectively, at identical initial geometries. The time evolution of the filamentary structure can be followed from the top to bottom panels. The complete structural evolution can be seen in Animation 1 in the Supplementary Information.

Figure 2 shows a typical simulated evolution of the junction at negative and positive tip potentials (left and right panels in Fig. 2, respectively). The structural development can be followed from the top to bottom panels. The initial asymmetrical arrangement, representing an STM tip - flat surface setup, develops in time very differently at opposite bias polarities. Figure 2(a,b) demonstrate that at the initial phase of the filament formation the region of the most intensive structural changes are located at the apex of the tip, where the electric field is the highest. At a negative tip potential the silver atoms are being deposited on the apex and a filament starts growing towards the bottom surface. Figure 2(c) illustrates a dendritic filament growth which is predominantly fueled by the oxidation of the silver atoms of the bottom electrode located under the filament. At a reversed polarity the tip gradually loses its apex leading to an effective shrinking while opposite filament growth starting from the flat surface toward the tip is taking place [Fig. 2(d)]. Figure 2(e,f) show the filament structures after establishing a metallic contact between the electrodes. Figure 2(e) demonstrates that the initial asymmetry of the contact is qualitatively preserved, while dendritic features in the filament and a shallow dip in the planar electrode also appear. These qualitative features are in remarkable agreement with *in-situ* experimental studies on the dynamics of nanoscale metallic inclusions in dielectrics^23^, where due to the high redox rates and ion mobility^1^ in stoichiometric Ag_2_S, qualitatively similar filament growth was observed.

**Figure 3:**
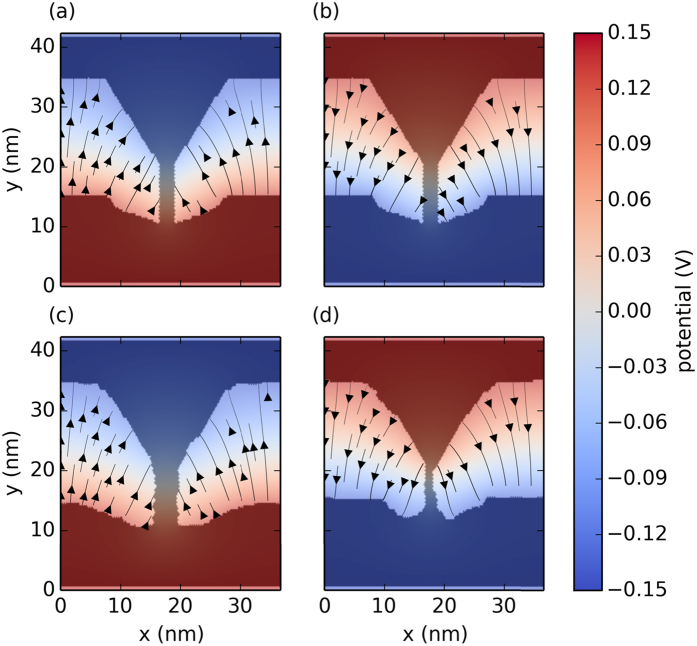
Time evolution of a metallic silver junction bridging asymmetric Ag electrodes under opposite bias voltages. The semi-transparent color map indicates the electrostatic potential and the stream lines visualize the electric field direction and magnitude across the Ag_2_S layer. The top panels show identical starting geometries. The bottom panels show the structure 1000 time steps later. The complete structural evolution is provided in Animation 2 in the Supplementary Information.

Next we study the relation of the preserved asymmetry after complete filament formation to the observed, robustly uniform polarity of the resistive switchings taking place between metallic OFF and ON states, corresponding to our experimentally investigated configuration [Fig. 1]. We performed simulations starting from simplified asymmetric filament boundaries representing an STM tip - flat surface setup as illustrated in Fig. 3(a,b). Figure 3(c,d) demonstrate demonstrate that under the influence of a negative (positive) tip bias the diameter of the filament increases (reduces) by time, in full agreement with the observed polarities of the set/reset transitions, underlining the fundamental role of the asymmetrical initial arrangement of the electrodes. The resulting electric field profiles reveal that the high field regions are located around the smallest cross-section of the contact, so that resistive switching can occur due to the increased probabilities of redox steps in the vicinity of the filament. We note that the migration of Ag^+^ ions in the mixed electronic/ionic conductor Ag_2_S matrix leads to self-doping of the material^26^. As the strongly doped Ag_2_S also participates in the electronic conduction near the filament, the effect of self-doping is expected to diffuse the boundary between the electronic and ionic conductor resulting in slightly modified electric field profiles as compared to those obtained by the simulations. This region, however, electrostatically becomes the part of the electrically conductive filament and can be effectively described as a metallic component. A more advanced, e.g. 3D simulation based on actual material parameters and taking the effect of self-doping explicitly into account could provide a more quantitative analysis.

**Figure 4:**
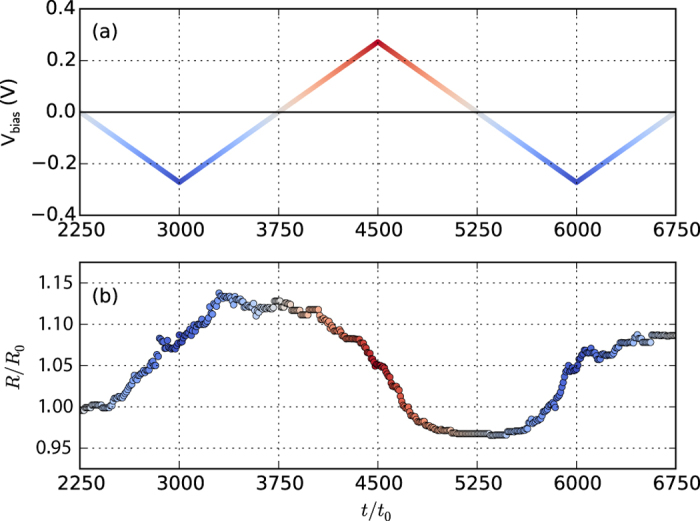
Simulated resistive switching upon a time-dependent bias voltage. (**a**) Time dependence of the bias voltage. (**b**) The corresponding resistance across the nanojunction. The complete structural evolution can be seen in Animation 2 in the Supplementary Information. *R*_0_ is a normalization factor accounting for the two-dimensional aspects of the simulation.

The simulated resistive states upon repeated biasing cycles are shown in Fig. 4. The two-terminal resistance is calculated from the bias voltage and the current through the contact which is 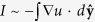 where the integration is over the horizontal cross-section of the two-dimensional plane. Due to its reduced dimensionality, our simple model is not expected to provide a quantitative agreement with the experimentally observed resistance values nor to account for the closing of the hysteresis loops upon a biasing cycle. Nevertheless, calculating the two-terminal resistance by taking, as a rude estimate, the bulk conductivity of silver and the narrowest (one-dimensional) cross-section of the junction into account, the basic qualitative features of the hysteretic I-V traces can be reproduced.

**Figure 5:**
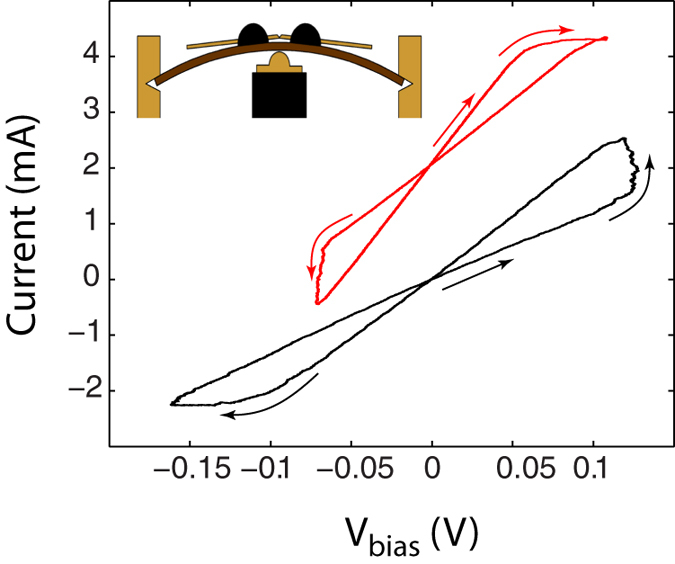
Representative I-V traces recorded in selected, independent junctions established in an MCBJ arrangement by a 2.5 Hz triangular *V*_drive_ voltage signal. The straight arrows indicate the random initial configurations measured after the creation of the junction at identical polarities. The hysteresis loops exhibit a random, uniform distribution of either clockwise or anti-clockwise direction. The upper (red) curve is vertically shifted for clarity. *R*_OFF_ = 77 Ω, *R*_ON_ = 45 Ω and *R*_S_ = 150 Ω (black trace), *R*_OFF_ = 45 Ω, *R*_ON_ = 25 Ω and *R*_S_ = 380 Ω (red trace). The inset illustrates the MCBJ setup.

In order to further verify the dominant role of the geometrical asymmetry in the polarity of the resistive switchings, we also performed experiments on sulfurized Ag-Ag junctions established in an MCBJ arrangement, as illustrated in the inset of Fig. 5. We note that our previous experiments^42^ utilizing the MCBJ technique for creating clean Ag-Ag atomic junctions at room temperature also reproduced the main features of the usually observed resistive switching behavior which were attributed to electromigration taking place finite bias. However, in the absence of an ionic conductor layer these individual characteristics were highly unstable in spite of the superior mechanical stability offered by the MCBJ technique^45^ over the one of an off-feedback STM setup.

Unlike in the STM setup, where the initial asymmetry of the junction is largely predetermined by the different shapes of the thin film and sharp tip electrodes, the controlled rupture of a uniform wire is expected to result in a randomly oriented local asymmetry at the apex of the nanojunction. In our present experiments stable, hysteretic I-V traces exhibiting comparable, metallic ON and OFF state resistances but opposite switching polarities were obtained after sulfurisation and controlled re-connection of the freshly created Ag-Ag junctions, as exemplified in Fig. 5. The statistical analysis of 10 identically prepared samples revealed a 50% probability of having a set/reset transition at a given voltage polarity independently from the applied bias during re-connection, providing an excellent agreement with our qualitative scenario. When a stable sequence of I-V measurements was followed by a complete rupture and re-connection of the electrodes, the polarity of the subsequent switchings were likely (>80%) to be identical to those obtained previously, indicating that up to a certain degree of re-establishment the asymmetry of the junction is mostly determined by the first rupture and is robust against repeated mechanical reconfigurations.

**Figure 6:**
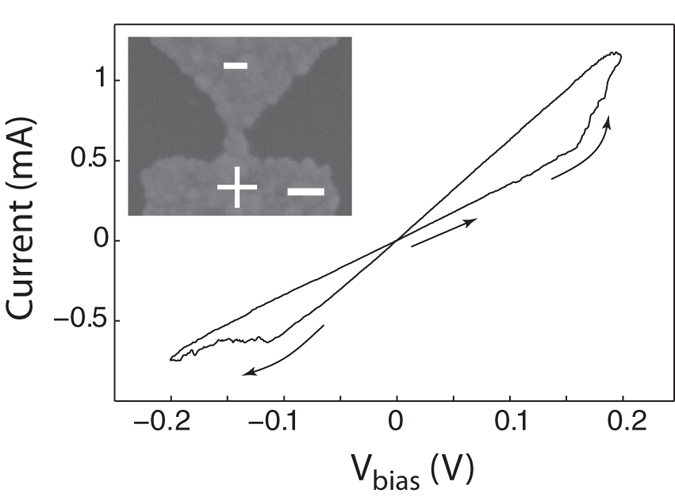
Representative I-V trace recorded by a 2.5 Hz triangular *V*_drive_ voltage signal in a planar Ag-Ag_2_S-Ag nanojunction created by electromigration and subsequent sulfurisation of an all-Ag lithographic structure. *R*_OFF_ = 279 Ω, *R*_ON_ = 155 Ω and *R*_S_ = 5 Ω. The inset shows the electron microscopy image of the device after performing the I-V measurements. The white scale bar in the lower right corner indicates 200 nm. The convention of the bias voltage polarity is also shown.

Based on the above findings we studied resistive switchings also in a series of prototype on-chip memory devices illustrated in the inset of Fig. 6. The structure mimicking the asymmetry of the STM arrangement was patterned by standard electron beam lithography on an amorphous, 140 nm thick SiN_x_ substrate. The 100 nm wide and 45 nm thick, electron beam evaporated silver channel connecting the electrodes was further reduced in its diameter by controlled electromigration in vacuum conditions^46^,^47^,^48^. This was followed by the opening of an approximately 1 nm wide gap at the narrowest cross-section of the silver channel situated presumably close to the apex of the triangular electrode, as suggested by the inset of Fig. 6. This nanogap was then exposed to vaporized sulfur at 60 °C at ambient pressure followed by the re-connection of the electrodes by triangular voltage signals up to 2 V amplitudes. An optimized, 3 minutes long sulfurisation time resulted stable, hysteretic I-V traces highly similar to those obtained by the previously discussed techniques, also reflecting identical switching polarities as exemplified in Fig. 6. This effect is attributed to the formation of a few 10 nm long region of Ag_2_S where filament formation and destruction can take place between the electrodes during memory operations. While the endurance of these very first proof of concept devices were in the order of a few 100 cycles we believe that, owing to their largely simplified structure and inherently high mechanical stability, the future optimization of the design and fabrication parameters is expected to enable the reliable application of such memory architectures.

**Figure 7:**
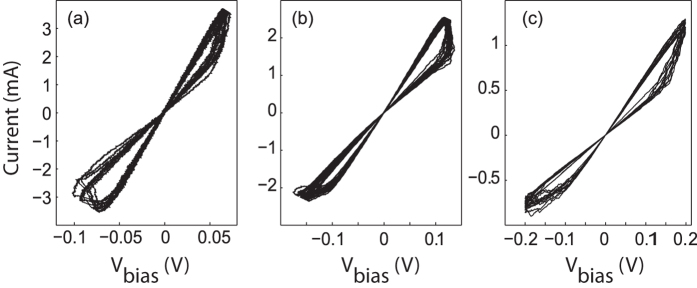
20 consecutive I-V traces acquired on stable nanojunction configurations established in an STM setup (**a**), MCBJ setup (**b**) and in a nanolithographic sample (**c**). *R*_OFF_ = 32.6 ± 3.3 Ω, *R*_ON_ = 18.2 ± 1.6 Ω (**a**), *R*_OFF_ = 77 ± 5 Ω, *R*_ON_ = 45 ± 1.8 Ω (**b**) and *R*_OFF_ = 279 ± 15 Ω, *R*_ON_ = 155 ± 5 Ω (**c**).

The reproducibility of the measured I-V traces at stable junction configurations are illustrated in Fig. 7(a–c) for STM, MCBJ and lithographically designed on-chip structures, respectively. The evaluated average *R*_ON_ and *R*_OFF_ values deduced from the curves shown in Fig. 7(a) and their ~10% relative standard deviation comply with those obtained in similarly established PtIr-Ag_2_S-Ag nanojunctions^40^ and are limited by the long-term mechanical stability of our off-feedback STM setup^18^. The superior mechanical stability of the MCBJ setup over the one of the off-feedback STM is reflected in the reduced relative standard deviations of the *R*_OFF_ and *R*_ON_ values in Fig. 7(b). While the STM and MCBJ setups were installed by implementing advanced isolation techniques against mechanical vibrations, the I-V measurements of the lithographic structures were carried out in a mechanically undamped vacuum chamber which was installed directly at the inlet of a turbomolecular pump. Yet, the lowest dispersions of the *R*_OFF_ and *R*_ON_ values were achieved in this setup [Fig. 7(c)], demonstrating the inherently high mechanical stability and robustness of our simplified on-chip design.

The long term stability of the resistance in unbiased structures is another key issue in realizing non-volatile memory applications. In accordance with the relatively high ionic mobility of Ag^+^ ions in the Ag_2_S matrix, atomic scale (<1 nm in diameter) Ag_2_S junctions exhibited both a random switching polarity^42^ and fast dissolution rates^27^. On the other hand, the high ionic mobility enabled reliable memory operations up to GHz switching speeds in Ag-Ag_2_S-PtIr nanojunctions with 2–5 nm in diameter^39^ where the long term stability of the nanofilaments were demonstrated up to 11 orders of magnitude larger time scales^18^. Moreover, since our present findings are explained in terms of pure asymmetry arguments, similar resistive switching behaviour in electrochemical metallization cells utilizing single component electrodes of other material systems characterized by different ionic diffusion rates is also envisioned.

In conclusion, we investigated stable resistive switchings in Ag-Ag_2_S-Ag nanojunctions lacking the conventionally employed inert electrode. Our experiments performed in the STM and MCBJ arrangements demonstrated that the polarity of the set/reset transitions are exclusively determined by the inhomogeneity of the local electric field, arising from the geometrical asymmetry present at the apex of the junction. Numerical simulations taking activated ion migration and redox reactions into account successfully reproduced the observed switching behavior also in the so-far-less-widely-investigated metallic regime. The simulations also reveal that the atomic re-arrangements responsible for the observed resistive switchings only involve a small amount of silver ions situated in the vicinity of the junction’s narrowest cross-section providing a key ingredient to ultrafast memory operation^39^. Our proof of principle experiments demonstrate the merits of lithographically designed Ag-Ag_2_S-Ag nanostructures as fast and highly integrable memory cells. Additionally, by further optimization of the nanometer-scale, planar on-chip design, an inherently high mechanical stability is envisioned, whereas the utilization of all-Ag electrodes makes the lithographical fabrication procedure uncomplicated.

## Methods

The first set of experiments utilized an 80 nm thick Ag layer deposited onto a Si substrate followed by a 5 minutes long sulfurisation performed at 60 °C resulting in a 30 nm thick stoichiometric Ag_2_S cap layer on the planar Ag electrode^49^. Nanometer-scale junctions were created between the Ag_2_S surface and a mechanically sharpened 99.99% pure Ag wire of 0.35 mm in diameter in STM geometry. Alternatively, Ag-Ag_2_S-Ag point contacts were also established by the controlled rupture of a 99.99% pure Ag wire with a diameter of 0.125 mm in the vacuum chamber of an MCBJ setup followed by a 20 minutes long *in-situ* sulfurisation carried out at 25 °C. During the electromigration process the junction was exposed to a series of 0.5 ms long voltage pulses of increasing amplitude ranging from 10 mV to a maximum of 300 mV. The sample’s resistance was monitored during the 100 ms dwell time between the pulses by acquiring low bias I-V traces. By repeating this method, the typical starting resistance of 50 Ω could be increased to 250 Ω. At this setpoint the bias was removed and due to a self-breaking mechanism^50^,^51^ an approximately 1 nm wide gap opened at the narrowest cross-section of the silver channel. During the acquisition of the current-voltage (I-V) characteristics the *V*_drive_ low frequency triangular voltage output of a data acquisition card was acting on the memristive junction and on a variable series resistor *R*_S_ as shown in Fig. 1(b). The device’s current was monitored by a current amplifier while the *V*_bias_ voltage drop on the junction was determined numerically as *V*_bias_ = *V*_drive_ − *I* · *R*_S_. All measurements were performed at room temperature.

## Additional Information

**How to cite this article**: Gubicza, A. *et al*. Asymmetry-induced resistive switching in Ag-Ag_2_S-Ag memristors enabling a simplified atomic-scale memory design. *Sci. Rep.* **6**, 30775; doi: 10.1038/srep30775 (2016).

## Supplementary Material

Supplementary Information

Supplementary Information

Supplementary Information
