# Supplementary material for: Asymmetry-induced resistive switching in Ag-Ag2S-Ag memristors enabling a simplified atomic-scale memory design
Source: Sci Rep. 2016 Aug 4;6:30775. doi: 10.1038/srep30775 (PMC4973259; doi:10.1038/srep30775)
Supplement: Supplementary Information [file srep30775-s1.pdf]

Supplementary information
Asymmetry-induced resistive switching in Ag-Ag_2S-Ag memristors enabling a simplified atomic-scale memory design

Agnes Gubicza,[1,2] Dávid Zs. Manrique,[3] László Pósa,[1,2] Colin J. Lambert,[3]
György Mihály,[1,2] Miklós Csontos[1,2*], and András Halbritter[1,2]
[1]*Department of Physics, Budapest University of Technology and Economics*
[2]*Condensed Matter Research Group of the Hungarian Academy of Sciences, 1111 Budapest, Budafoki ut 8, Hungary*
[3]*Physics Department, Lancaster University, Lancaster, UK*

In order to qualitatively investigate the silver filament growth in Ag-Ag_2S-Ag structures we performed atomic scale two-dimensional lattice based simulations. The simulation is implemented on an equilateral triangular lattice where a site can represent an empty site, a silver atom or a mobile silver ion. The presence of sulphide ions is taken into account as a screening medium reducing the range of the silver ion interactions to nearest neighbor. The microscopic development is driven by room temperature ionic diffusion and redox processes, the latter taking place at the electrode surfaces. Similar filament growth simulations have been done before in Ref. 1 utilizing different theoretical models.

A typical equilateral triangular lattice used in the simulations can be recognized in Fig. S1 where the black dots denote silver atoms, the red dots represent silver ions. The lattice exhibits periodic boundary conditions along the horizontal direction while the vertical boundaries are terminated by two layers of silver atoms set to V and zero potentials on the top and bottom, respectively. The lattice constant is set to a=3.85 which approximately corresponds to one real atom per site. The electrostatic potential on each site is computed by solving the Poisson's equation $\nabla(\epsilon_r \nabla u) = -\rho/\epsilon_0$ on the lattice. The charges of the silver ions are considered to be screened, therefore they are excluded from the electrostatic calculation. $\epsilon_r = 1$ is set to zero outside, whereas inside the silver $\epsilon_r = 1 - i1.25 \times 10^5$ is applied. The surface charge density is computed from the potential as $\triangle u = -\rho/\epsilon_0$.

The time development is performed either by moving some of the silver ions or atoms to their neighboring empty site or by simulating a redox reaction, in which silver ions and atoms located at an electrode surface are exchanged. First the electrostatic potential is computed in each time step. This is followed by the calculation of a transition probability for each possible change. Finally the changes are executed with the calculated probabilities. The transition probability of a silver ion at site k_+ moving to its neighboring empty site $k_\circ$ is computed as [2]

$$w^{\text{diff}}_{k_+,k_\circ} = \min\left(1, \frac{\Delta t}{\tau_+} e^{-\frac{\Delta E^{\text{diff}}_{k_+,k_\circ}}{k_B T}}\right),$$

where $\Delta E^{\text{diff}}_{k_+,k_\circ}$ is the energy change of the move, $1/\tau_+$ is the attempt frequency of the silver ion to jump and Δt is the duration of the time steps. The $w^{\text{diff}}_{k_\bullet,k_\circ}$ transition probability of a silver atom jumping from site $k_\bullet$ to its neighboring empty site $k_\circ$ is computed similarly using $\Delta E^{\text{diff}}_{k_\bullet,k_\circ}$ and $1/\tau_\bullet$. The transition probability for a redox step, where a surface silver atom on site $k_\bullet$ is oxidized and a surface silver ion on site $k_+$is reduced, is computed as

$$w^{\text{redox}}_{k_\bullet,k_+} = \min\left(1, \frac{\Delta t}{\tau_{\text{redox}}} e^{-\frac{\Delta E^{\text{ox}}_{k_\bullet} + \Delta E^{\text{red}}_{k_+}}{k_B T}}\right),$$

where $\Delta E^{\rm ox}_{k_\bullet} + \Delta E^{\rm red}_{k_+}$ is the energy change due to the oxidation and reduction on site $k_\bullet$ and site k_+, respectively, and $1/\tau_{\rm redox}$ is the redox reaction rate. The Δt duration of the time steps is chosen such that the typical transition probability is much smaller than 1. The energy changes are calculated as

$$\Delta E^{\rm diff}_{k_+,k_\circ} = \tilde{\mu}_{k_\circ}(Ag^+) - \tilde{\mu}_{k_+}(Ag^+)$$

$$\Delta E^{\rm diff}_{k_\bullet,k_\circ} = \tilde{\mu}_{k_\circ}(Ag) - \tilde{\mu}_{k_\bullet}(Ag)$$

$$\Delta E^{\rm ox}_{k_\bullet} = \tilde{\mu}_{k_\bullet}(Ag^+) - \tilde{\mu}_{k_\bullet}(Ag) + \tilde{\mu}^e_{k'_\bullet}$$

$$\Delta E^{\rm red}_{k_+} = \tilde{\mu}_{k_+}(Ag) - \tilde{\mu}_{k_+}(Ag^+) - \tilde{\mu}^e_{k'_+}$$

where the electrochemical potential of a silver ion at site k is

$$\tilde{\mu}_k(Ag^+) = \gamma_{++} n^+_k + \gamma_{+\bullet} n^\bullet_k + |e| u_k,$$

where n^+_k and $n^\bullet_k$ are the numbers of silver ion and atom neighbors of site k, respectively, γ_{++} and $\gamma_{+\bullet}$ are the interaction energies, $|e|$ is the charge of the silver ion and u_k is the potential at site k. The electrochemical potential for the silver atom is

$$\tilde{\mu}_k(Ag) = \gamma_{+\bullet} n^+_k + \gamma_{\bullet\bullet} n^\bullet_k,$$

where the $\gamma_{\bullet\bullet}$ is the interaction energy between silver atoms. The sites $k'_\bullet$ and k'_+ denote neighboring silver sites to $k_\bullet$ and k_+, respectively. They are chosen to provide the maximum probability for the given redox reaction. The electron's electrochemical potential on a surface site is calculated as [3]

$$\tilde{\mu}^e_k = E_F - |e| u_k - \frac{\rho_k/|e|}{g(E_F)},$$

where ρ_k is the charge density at the surface site k and $g(E_F)$ is the surface density of states in silver. The structural evolution of the contact is determined by the locations of the redox processes. The above formulae of the chemical potential imply that the typical locations of the redox reactions are determined by the average numbers of neighbors i.e., the ion concentration, and by the surface charge density. The numbers of neighbors are weighted with the interaction energies, therefore this contribution is coupled to the energetics of the diffusion process, whereas the surface charge density terms depend on the metal properties.

The typical parameter values utilized in the simulations were $\tau_+/\tau_{\rm redox}$=0.001, $\tau_\bullet/\tau_{\rm redox}$=0.01, γ_{++}/k_BT=3, $\gamma_{+\bullet}/k_BT$=2, $\gamma_{\bullet\bullet}/k_BT$=-20, $k_BTg(E_F)a^3\sqrt{3} = 3 \times 10^{-5}$, eV/k_BT=10, and the concentrations of mobile silver ions, $c \approx 1/3$. Adjusting the parameter values two extreme scenarios may take place: the initial structure either explodes or no significant change occurs during the simulated timescale. Trying different parameter sets to avoid such extreme outcomes, we found that resistive switching is a robust phenomenon in the simulated memristor structures which does not require fine tuning of the parameters. Increasing the voltage bias or reducing the $\gamma_{\bullet\bullet}/k_BT$ strength of the silver-silver interactions can lead to more dendritic evolvement and also the structure more likely explodes to a random mixture of atoms and ions. The $k_BTg(E_F)a^3\sqrt{3}$ parameter provides a means to increase the probability of redox reactions without tuning the diffusion parameters.

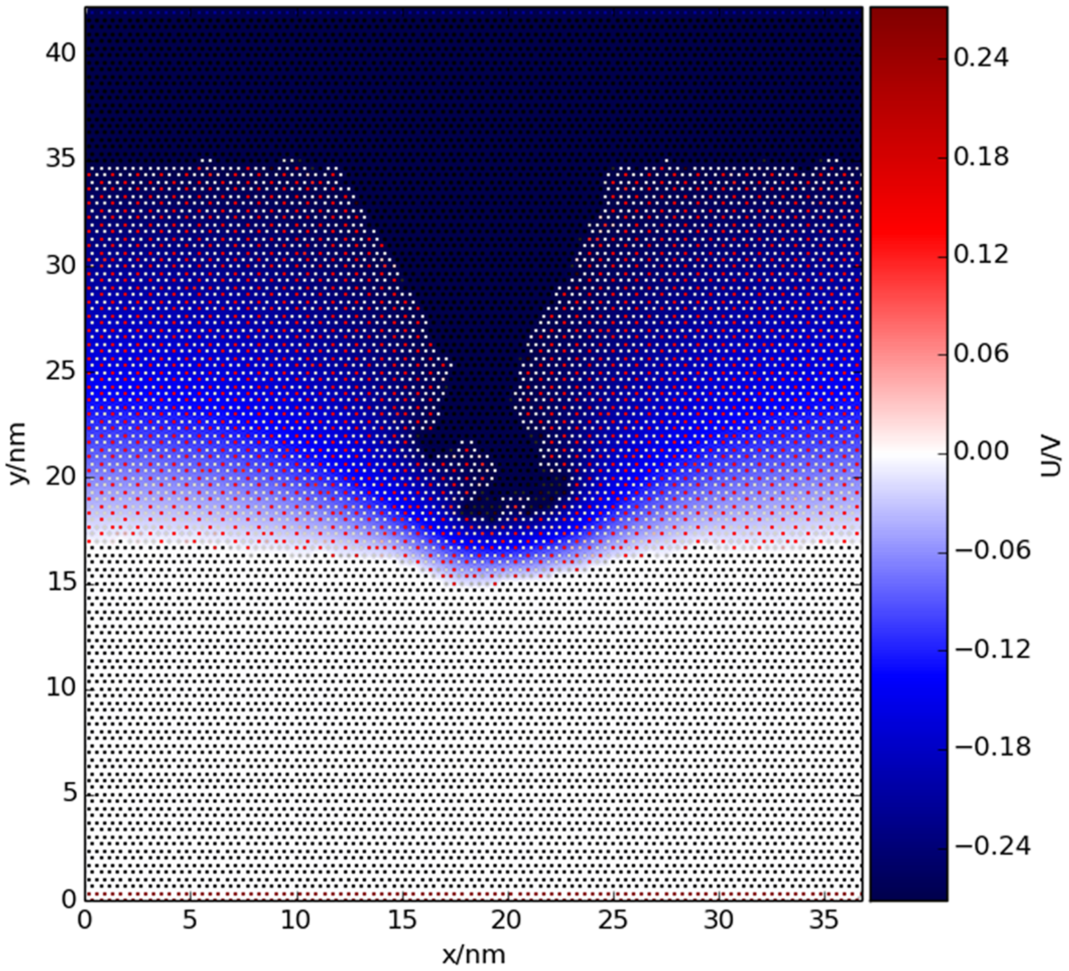

Figure S1. Snapshot of initial silver filament formation within the Ag_2S layer at an asymmetric tip versus flat surface arrangement of the Ag electrodes. The semi-transparent color map indicates the electrostatic potential. The black (red) dots represent Ag atoms (Ag^+ ions) The grey dots denote empty sites.

A snapshot of the electrostatic potential and surface charge density maps corresponding to a dendritic filament formation are displayed along with the underlying lattice in high resolution in Figs. S1 and S2, respectively. Two animation files are also added to the present Supporting information. Animation 1 shows the structural evolution during silver filament formation in an asymmetrical tip - flat surface electrode arrangement, as explained via the snapshots displayed in Fig. 2 of the main text. Animation 2 shows the modulation in the width of a completed metallic filament having asymmetrical boundaries

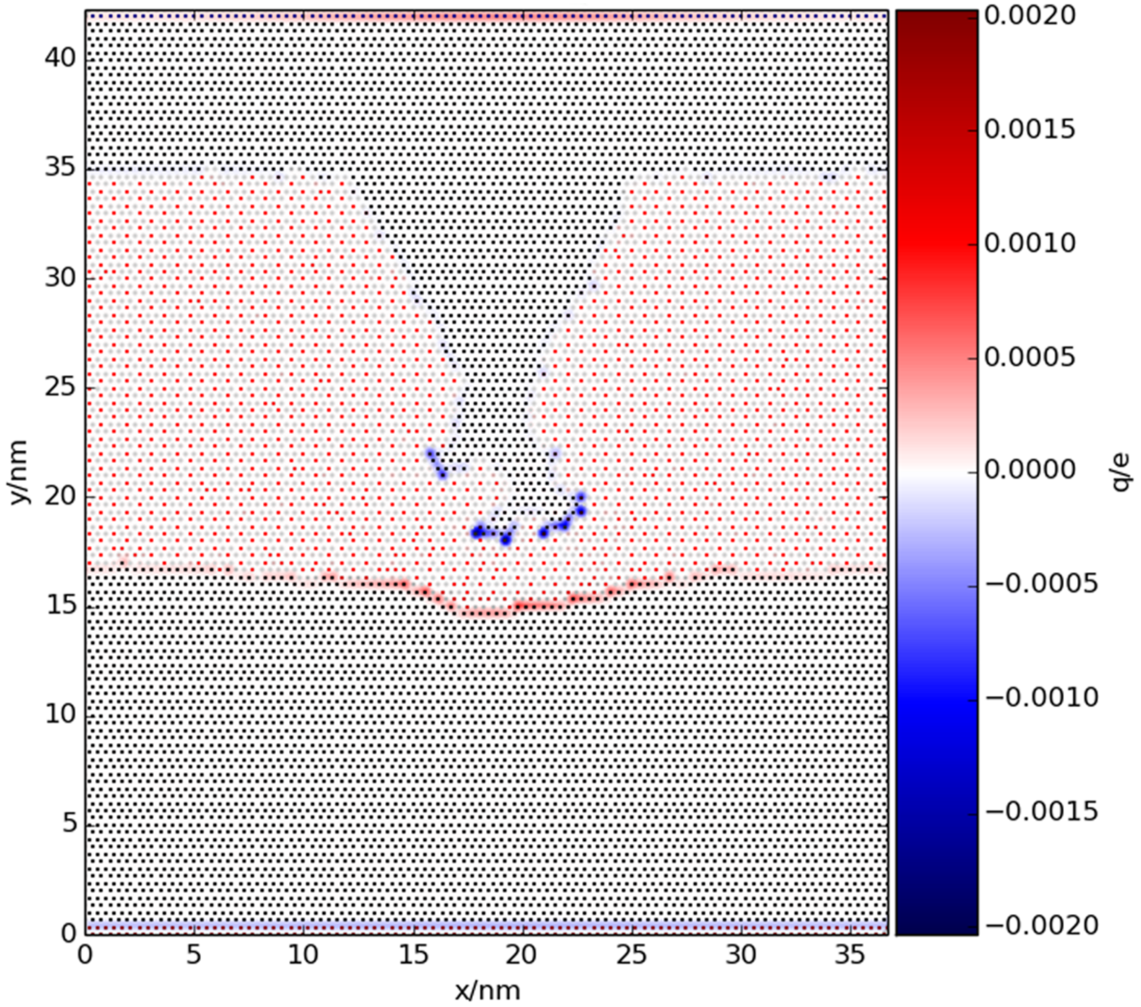

Figure S2. Color map of the surface charge density in the arrangement of Fig. S1. The black (red) dots represent Ag atoms (Ag^+ ions). The grey dots denote empty sites.

(as seen in Fig. 3 of the main text) under bias voltages of alternating sign.

[1] Zhang, Y., Islam Mou, N., Pai, P. & Tabib-Azar, M. Quantized current conduction in memristors and its physical model. *IEEE Sensors Proceedings*, 819-822 (2014).

[2] Schmittmann, B. & Zia, R. K. P. Statistical mechanics of driven diffusive systems. In Domb, C. & Lebowitz, J. L. (eds.) *Phase Transitions and Critical Phenomena* vol. 17 (Academic Press, London, 1995).

[3] Bernard, M.-O., Plapp, M. & Gouyet, J.-F. A mean-field kinetic lattice gas model of electrochemical cells. *Phys. Rev. E* **68**, 011604 (2003).
